# Nurse staffing, direct nursing care hours and patient mortality in Taiwan: the longitudinal analysis of hospital nurse staffing and patient outcome study

**DOI:** 10.1186/1472-6963-12-44

**Published:** 2012-02-20

**Authors:** Yia-Wun Liang, Wen-Yi Chen, Jwo-Leun Lee, Li-Chi Huang

**Affiliations:** 1 Department of Senior Citizen Service Management, National Taichung University of Science and Technology, No.193, Sec 1, Sanmin Road, Shi, Taichung 40343, Taiwan (Republic of China; 2 Department of Leisure Business Management, Nan Kai University of Technology, No.568, Chung Cheng Road, Tsao Tun County, Nantou 54243, Taiwan (Republic of China; 3 Department of Senior Citizen Service Management, National Taichung University of Science and Technology, No.193, Sec 1, Sanmin Road, Shi, Taichung 40343, Taiwan (Republic of China; 4 Department of Nursing, China Medical University, No.91, Hsueh-Shih Road, Taichung 40401, Taiwan (Republic of China

**Keywords:** Nurse staffing, Direct nursing care hours, Patient outcome, Mixed effect logit model

## Abstract

**Background:**

Studies over the past decades have shown an association between nurse staffing and patient outcomes, however, most of these studies were conducted in the West. Accordingly, the purpose of this study aimed to provide an overview of the research/evidence base which has clarified the relationship between nurse staffing and patient mortality of acute care hospital wards under a universal health insurance system and attempted to provide explanations for some of the phenomena that are unique in Taiwan.

**Methods:**

Through stratified random sampling, a total of 108 wards selected from 32 hospitals in Taiwan were collected over a consecutive seven month period. The mixed effect logit model was used to explore the relationship between nurse staffing and patient mortality.

**Results:**

The medians of direct-nursing-care-hour, and nurse manpower were 2.52 h, and 378 persons, respectively. The OR for death between the long direct-nursing-care-hour (> median) group and the short direct-nursing-care-hour (≦median) group was 0.393 (95% CI = [0.245, 0.617]). The OR for death between the high (> median) and the low (≦median) nurse manpower groups was 0.589 (95% CI = [0.381, 0.911]).

**Conclusions:**

Findings from this study demonstrate an association of nurse staffing and patient mortality and are consistent with findings from similar studies. These findings have policy implications for strengthening the nursing profession, nurse staffing, and the hospital quality associated with nursing. Additional research is necessary to demonstrate adequate nurse staffing ratios of different wards in Taiwan.

## Background

The Institute of Medicine's (IOM) report "Keeping Patients Safe: Transforming the Work Environment of Nurses" indicates that low nurse staffing in hospitals is one of the causes contributing to 98,000 preventable deaths each year in the United States [1]. Physicians have also cited inadequate nurse staffing as a major impediment to providing high quality hospital care [2]. Because of an aging nurse workforce, problems with retention and difficulty with recruiting young people into the field, coupled with the severe nursing shortage seemingly will continue to exist in the U.S. [3]. Furthermore, the U.S. nursing shortage is projected to grow to 260,000 registered nurses (RN) by 2025. A shortage of this magnitude would be twice as large as any nursing shortage experienced in U.S. since the mid-1960s [4]. A large majority of hospitals in Taiwan are also short of nurses and the nurses shortage has been especially serious in recent years and has led to the closing of wards at some hospitals according to the results of a National Union of Nurses' Associations (NUNA) survey [5].

Past research has shown that hospitals with low or inadequate nurse staffing levels have poorer patient outcomes, thus compromising patient care. Decreased nurse staffing, specifically RNs had an association with patient safety and increased adverse events such as pneumonia [6,7], urinary tract infections (UTI) [6,8], pressure ulcers [6], medication errors [9], patient mortality [10-14], patient falls [13,14], longer average length of stay (ALOS) [15], and unplanned extubations [16]. In addition, higher total nursing hours of care are associated with lower rates of patient falls [14], pressure ulcers, patient complaints and mortality [17]. A higher proportion of hours provided by RNs was found to be associated with a shorter ALOS [18], and less incidents of UTI, upper gastrointestinal bleeding, pressure ulcers, patient complaints and medication errors [17], pneumonia [19,20], and shock or cardiac arrest with failure to rescue [21].

Taiwan, an island nation with 23 million people, implemented a single-payer, single-administration universal health insurance program in 1995. As with other nations, Taiwan has experienced escalating health care expenditures. Accordingly, the government has adopted a prospective payment system to contain growing medical expenditures. Since 2002, many hospitals, facing financial pressures from the hospital global budget payment system (with an annual fixed budget reimbursed by hospitals based on fee-for-service), have undergone radical restructuring of their operations. Hospital administrators reorganized patient care services by hiring more unlicensed assistive nursing personnel (at salaries lower than an RN) or casual nurses to replace RNs [22]. Nevertheless, it is difficult to increase nurse staffing levels, even with the replacement of RNs with less costly staff members. Factors contributing to the difficulties of increasing nurse staffing levels include working conditions, the gap between the number of available positions and the number of qualified RNs willing to fill them [23,24], job dissatisfaction and burnout resulting from the nursing shortage[24]. Furthermore, in Taiwan, nurses are leaving the profession because of involvement in functions other than patient care on the hospital floor such as utilization management and nursing informatics [25]. This is evident from a recent study indicating an average vacancy rate of 28% in Taiwan [22].

It should be noted that Taiwan has legislation mandating minimum nurse-to-patient ratios for hospitals. Furthermore, families usually accompany and stay with patients in the hospital, but this minimum ratio (1 nurse: 8 patients) was not put into practice until 2009 and it is only valid for the daytime shifts. Therefore, hospitals might have adequate nurse staffing during daytime shifts but fail to meet the staffing ratios at nights leading to 16 and 22 patients per nurse for second and third shift, respectively [22]. The mean patient load across all staff nurses is considered to be higher than those mandated ratios enacted by California and Australia [26].

Despite mounting evidence that higher nurse-to-patient ratios and a greater number of RNs in the skill mix reduce adverse patient outcomes in the U.S. [6-13,15,17,18,21], investigations have provided little substantial or analytic consideration to explain the relationship between nurse staffing and patient outcomes under the Taiwan National Health Insurance system. Inadequate nurse staffing levels seem to be the number one concern of Taiwanese nurses today and, for too long, hospital administrators have viewed nursing as an expense rather than human capital in delivering quality, cost-effective care [22], leading nurses who begin their careers in hospitals to frequently leave for other positions causing the professional lifespan of low-level nurses to have plummeted to around seven years [5].

Taiwan has a differently organized healthcare system (e.g., single payer system, high insured rate, involvement of the family member when hospitalized) compared to other countries, that may result in different findings. However, evidence for a universal healthcare system is limited. Accordingly, this study aimed to explore the effect of acute care hospital nurse staffing on patient mortality in Taiwan. To the authors' best knowledge, no research has previously examined this issue based on nationally-representative hospital data in Taiwan. Furthermore, this study can provide an overview of the research and evidence base which has clarified the relationship between nurse staffing and patient mortality of acute care hospital nursing units under a differently organized healthcare system. The aim of this study is to answer the question, "What are the effects of acute care hospital nurse staffing on patient mortality?" In response to this research question, we tried to empirically test the hypothesis that an increase of hospital nurse staffing will reduce patient mortality. It is hoped that with this information, policymakers will be able to make more informed decisions in terms of nurse staffing level adjustments, while optimizing quality of care and improving nurse satisfaction.

## Methods

### Sample and procedure

The data used in this study were obtained from the 2008 Hospital Nurse Staffing and Patient Outcomes Project sponsored by the Taiwan Department of Health (DoH). The sample for the study was drawn from the populations of medical wards, surgical wards, comprehensive wards, and intensive care units in 421 accredited Western-medicine hospitals in Taiwan at the end of 2006, among a total of 556 Western-medicine hospitals (88 public hospitals and 468 private hospitals). This study excluded psychiatric hospitals (38 hospitals) and hospitals without accreditation (79 hospitals). Through stratified random sampling, we selected 69 hospitals (about twice sample size as we need) because the expected participating rate was anticipated to be low. The final sample size was 32 hospitals resulting in a 46.4% response rate. We collected data from a total of 108 wards within 32 hospitals over a consecutive seven month period. Hospitals participating in DoH projects refused to provide patient-level data, so we used "ward" as our unit of analysis. Monthly data were collected on site from July 1^st ^2008 to January 31^st ^2009, which resulted in a total of 756 observations.

### Instrument and variable definition

#### Instrument

The research design of this study was a longitudinal panel survey. The survey instrument used was a questionnaire, consisting of hospital characteristics, ward characteristics, actual ward nursing manpower, patient demographics, and patient outcomes. This questionnaire was developed based on past studies [10,21,27] and expert opinion. All questions were answered by nurse managers in each ward on the basis of clinical standards set forth by the Taiwan Hospital Accreditation Association.

#### Variables definition

The conceptual model for this study emphasizes a traditional relationship between nursing hours and patient mortality which may be related to both patient and provider characteristics. Accordingly, the study includes patient factors and hospital organizational factors that may influence the effect of nurse staffing on patient outcomes. All variables selected for our analyses were not decided by the statistical method chosen for our study but were chosen by previous (both theoretical and empirical) studies in determining that examined factors influencing the patient outcome. All variables used in this study were introduced as follows:

Given that each hospital ward under study had seen many patients over a one-month period, the dependent variable was the binary outcome variable (Y). If any or at least one death is observed over a one-month period, Y is set equal to one and if no death was observed, Y is set equal to zero. The patient mortality considered both in-hospital and 30-day mortality together, because estimating hospital-related mortality based only on in-hospital deaths may be influenced by hospital discharge practices [28] and could result in lower in-hospital mortality rates that are independent of the quality or effectiveness of hospital care. Out-of-hospital mortality information was obtained from death records by linking unique patient identifiers. Independent variables included labor factors (such as direct nursing care hours, and nurse manpower), and capital factors (such as hospital scale), and control variables (severity of disease and time variable). The direct nursing care hours represent average nursing hours that contribute to direct care to patients per day over a one-month period, and were calculated by the formula (1) [29]:

(1) $$\text{DNCH}=\frac{\text{Working}\;\text{hours}\;\times \;\text{Number}\;\text{of}\;\text{Nurses}}{\text{Beds}\;\times \;\text{Occup}\;\text{ancy}\;\text{rate}}$$

The numerator in formula (1) represents the total working hours contributed by total nurse staffing on the observed ward. The denominator represents the total number of patients the combined nurse staffing on the observed ward was responsible for. Currently, nurses are in fact multi-task care givers, so the direct nursing care hours is a measure of the quality of nurse manpower input that used to care for patients. Higher average direct nursing care hours allow nurses time for appropriate assessment of patients and their needs and an initiation of suitable interventions. Therefore, higher direct nursing care hours is hypothesized have better equality or lower patient mortality.

Labor factors included two dummy variables to distinguish different manpower inputs. The first dummy variable was defined as using the median direct nursing care hours as a cut-off point, and the study participants were differentiated into two groups: long direct-nursing-care-hour group (coded as 1) and short direct-nursing-care-hour group (coded as 0). The second dummy variable was defined as using the median number of nurses staffing to hospital as a cut-off point, and the study participants were differentiated into two groups: high nurse manpower group (coded as 1) and low nurse manpower group (coded as 0). All nurse manpower in this study was represented by either registered or licensed nurses.

Capital factors included three dichotomized variables to distinguish different hospital capital factors. The first dichotomized variable was defined as using the median number of hospital beds as a cut-off point, and the study participants were differentiated into two groups: large scale hospital (coded as 1) and small scale hospital (coded as 0). In addition, we used two dichotomized variables to distinguish three different types of hospitals: medical center, regional hospital, and district hospital. Typically, medical centers have the largest scale and the most capital input, regional hospitals are intermediate in scale and capital input, and district hospitals have the smallest scale and least capital input.

Control variables consist of different types of wards, the average patient age, the ALOS in the observed ward, and time variable. Age, and ALOS have been served as proxies for severity of illness [30]. The median age was used to distinguish the study participants into two groups: the older group (coded as 1) and the younger group (coded as 0), and the median of ALOS was used to distinguish the study participants into two groups: the long ALOS group (coded as 1) and the short ALOS group (coded as 0). Four types of hospital inpatient units (internal medicine ward, surgical ward, comprehensive ward, and intensive care unit) were also used to control for different severities of illness. A patient's illness is considered more complicated than their counterparts, if they are hospitalized in the intensive care unit. Therefore, we take 'intensive care unit' as a reference group to indicate a different complexity of illness in the observed inpatient unit. Finally, the time variable, describing months since the start of the study, coded as an increasing index number from one to seven, was used to control for longitudinal effects or trend over time.

### Statistical analysis

#### Mixed effect logit model

We used a mixed effect logit model to investigate the relationship between patient mortality and nurse staffing (such as direct nursing care hours and nurse manpower) as well as other independent variables (such as hospital scale and severity of illness). The reason to choose the mixed effect model for our statistical analyses is because our data were collected in a form of panel data (i.e., the same hospital ward was observed over a seven consecutive month period). These data measured from the same ward were correlated.

It should be noted that the mixed effect logit model is a nonlinear model, and the independent variables are categorical. Therefore, we took the natural exponential value of the estimated coefficient so that the odds ratio (OR, defined by a ratio of two odds) for incidence of death between the target group and the reference group could be obtained. The mixed logit effect model was estimated by MLE type method proposed by Greene [31].

### Model selection and goodness of fit

The intra-class correlation (ICC) criterion was used to decide whether the mixed effect logit model is relevant to our cluster data. The ICC is an indication of the proportion of variance in occurrence of death among hospital wards. Cohen [32] suggests that if the ICC is higher than 5.9%, the effect of cluster individuals (repeat panel data) within the same aggregate unit (hospital wards) in estimating the regression model cannot be neglected. It follows that the mixed logit model should be used for our analyses. In addition, the Hosmer-Lemeshow test (HL diagnostic statistic follows chi-square distribution of eight degree of freedom) [33] was used to test whether the selected mixed effect logit model are as good as the saturated model.

## Results

### Descriptive statistics

A total of 108 wards selected from 32 hospitals (3 medical centers, 18 regional hospitals, and 11 district hospitals) in Taiwan completed the survey. The sample roughly represented all hospitals within a proportional allocation in Taiwan. The descriptive statistics for all variables are listed in Table 1. As shown in Table 1, during our observation period, the average death cases and its median were 4.36, and 2.00, respectively. Approximately 68% of hospital wards observed at least one death during our study period. The average direct nursing care hours and its median were approximately 4.95, and 2.52 h, respectively. The mean (median) age of patients was approximately 60 (59) years. The mean (median) ALOS was 7.59 (6.80) days. In our sample, ward samples distributed to internal medicine wards, surgical wards, comprehensive wards, and intensive care units were 34%, 25%, 12%, and 29%, respectively. Our samples distributed to district hospitals, regional hospitals, and academic medical centers were 31%, 58%, and 11%, respectively. The mean (median) of hospital beds were 638 (645). Finally, the mean of nurses staffing to hospitals and its median were 420, and 378, respectively.

**Table 1:** Descriptive Statistics

Variables	Classification	Sample Size	%
Death Cases	*Mean*(*Median*) ± *SD*	*4.36*(*2.00*) ± *6.10*	
Occurrence of Death	Death = 1	517	68.39
	Others = 0	239	31.61
Direct Nursing Care Hours (DNCH)	*Mean*(*Median*) ± *SD*	*4.95*(*2.52*) ± *4.20*	
Age	*Mean*(*Median*) ± *SD*	*59.77*(*59.41*) ± *9.29*	
Average Length of Stay (ALOS)	*Mean*(*Median*) ± *SD*	*7.59*(*6.80*) ± *3.82*	
Type of Wards	Internal Medicine Ward	259	34.26
	Surgical Ward	189	25.00
	Comprehensive Ward	91	12.04
	Intensive Care Unit	217	28.70
Type of Hospitals	Medical Center	84	11.11
	Regional Hospital	441	58.33
	District Hospital	231	30.56
Hospital Scale	*Mean*(*Median*) ± *SD*	*638.02*(*645.00*) ± *273.36*	
Nurse Manpower	*Mean*(*Median*) ± *SD*	*420.11*(*378.00*) ± *233.56*	

### Model selection and goodness of fit

Table 2 displayed the estimation results for the mixed effect logit model. We found that the ICC generated by the mixed effect logit model with a random intercept was 65%. A 65% ICC means that 65% of variance in the occurrence of death comes from the variation across observed hospital wards. It seems that the mixed effect logit model may be an appropriate model to analyze our data since a 65% of ICC is well above 5.9%, a criterion suggested by Cohen [32]. In addition, the ICC derived from the final fitted model dropped sharply from 65% to 39%. Furthermore, the Hosmer-Lemeshow(HL) diagnostic statistic(HL = 8.20 with *p*≧0.1) showed that the mixed effect logit model presented in Table 2 was as good as the saturated model. Therefore, our analyses proceed with the mixed effect logit model.

**Table 2:** Empirical Results for the Mixed Effect Logit Model

	Coefficient (z value)	OR [95% CI]	
Direct Nursing Care Hours	-0.935	0.393	
(if DNCH > Median)	(-4.05)**	[0.245, 0.617]	
Nurse Manpower	-0.530	0.589	
(if Nurses > Median)	(-2.38)*	[0.381, 0.911]	
Medical Center	0.017	1.017	
	(0.05)	[0.521, 1.988]	
Regional Hospital	0.761	2.140	
	(2.95)**	[1.291, 3.546]	
District Hospital (Reference Group)	------	------	
Hospital Scale	0.100	1.105	
(if # of Beds > Median)	(0.47)	[0.731, 1.670]	
Age	0.597	1.816	
(if > Median)	(3.27)**	[1.270, 2.598]	
Length of Stay	0.696	2.007	
(if LOS > Median)	(4.08)**	[1.436, 2.804]	
Internal Medicine Ward	-2.245 (-7.97)**	0.106 [0.061, 0.184]	
Surgical Ward	-3.785 (-11.03)**	0.023 [0.012, 0.044]	
Comprehensive Ward	-1.845 (-5.40)**	0.158 [0.081, 0.309]	
Intensive Care Unit (Reference Group)	------	------	
Time Variable	-0.004 (-0.11)	------	
Constant	2.512 (5.97)**	------	
ICC with intercept only		65.30%	
ICC with final fitted model		38.58%	
	H_0_: Selected Model vs. H_A_: Saturated Model		
Hosmer-Lemeshow Test	$\chi_{\text{df}=8}^{2}=8.20$*p *value = 0.41		

"**" and "*" represent 1% and 5% significance level, respectively

### Mixed effect logit model

The results of the association between labor factors and patient mortality were as expected. We found that the estimated coefficient of the direct nursing care hours was statistically negatively at a 1% significance level. The OR for the incidence of death between the long direct-nursing-care-hour (> median) group and the short direct-nursing-care-hour (≦median) group was 0.393 (95% CI = [0.245, 0.617]). These results indicated that the direct nursing care hours had a significantly negative association on the incidence of death. The risk of the incidence of death in the long direct-nursing-care-hour group was much lower than that in the short direct-nursing-care-hour group. Moreover, the estimated coefficient of the nurse manpower was statistically negative at a 5% significance level. The OR for the incidence of death between the high (> median) and the low (≦median) nurse manpower groups was 0.589 (95% CI = [0.381, 0.911]). This result indicated that the nurse manpower had a significantly negative association with the incidence of death. The risk of the incidence of death in a high nurse manpower group was much lower than that in a low nurse manpower group.

With respect to the association between capital factors and patient mortality, only the estimated coefficient of regional hospitals attained statistical significance (at 1% significance level). The OR for the incidence of death between regional hospitals and district hospitals was 2.140 (95% CI = [1.291, 3.546]). This result indicated that the risk of the incidence of death in regional hospitals was much higher than that in district hospitals. Note that the estimated coefficients of hospital scale (measured by number of beds) and medical center did not reach any statistical significance. This finding suggested that the reduction of patient mortality may rely less on hospital scale but more on nursing care (such as direct nursing care hours).

As expected, we found that the estimated coefficient of age was statistically positive at a 1% significance level. The OR for the incidence of death between the older (> median) and the younger (≦median) group was 1.816 (95% CI = [1.270, 2.598]). These results indicated that age had a significantly positive association with the incidence of death. The risk of the incidence of death in the older group was much higher than that in the younger group. In addition, the sign of the estimated coefficient of ALOS was statistically positive at a 1% significance level. The OR for the incidence of death between the long (> median) and the short (≦median) ALOS groups was 2.007 (95% CI = [1.436, 2.804]). These results indicated that ALOS had a significantly positive association with the incidence of death. The risk of the incidence of death in the long ALOS group was much higher than that in the short ALOS group.

Moreover, the estimated coefficients of internal medicine wards, surgical wards, and comprehensive wards were statistically negative at a 1% significance level, and their corresponding ORs for the incidence of death were 0.106 (95% CI = [0.061, 0.184]), 0.023 (95% CI = [0.012, 0.044], and 0.158 (95% CI = [0.081, 0.309]), respectively. These results indicated that the risk of the incidence of death in intensive-care units was much higher than in internal medicine wards, surgical wards, and comprehensive wards. The sign of estimated coefficient of time variable was negative, but it did not reach any statistically significance level.

## Discussion

After controlling for the hospital level factors, nurse staffing (direct nursing care hours and nurse manpower) was found to be significantly associated with patient mortality. The risk of the incidence of death in a long direct-nursing-care-hour (a high nurse manpower) group was found to be much lower than that in a short direct-nursing-care-hour (a lower nurse manpower) group after controlling for severity of illness and hospital characteristics. Our results provided further evidence of the importance of nurse staffing to high quality patient outcomes similar to findings in three other studies which examined the association between total nurse hours per patient day and hospital related mortality two at hospital level [21,27] and one at the unit level [17]. The reduction in mortality at discharge is not unexpected because 25% of the samples were surgical patients who were recovering from the immediate association of surgery and anesthetic. However, it is important to note that the average direct nursing care hours (from Table 1) were 4.95 per patient day, only approximately 21% of a full patient care day (24 h). That is apparently inadequate based on the evidence of positively correlation between the direct nursing care hours and patient mortality. Our results emphasized the fact that the shortage of direct nursing care hours to patients would jeopardize the quality of care in acute care hospitals in Taiwan.

In addition, nursing care affects the outcomes of hospitalized patients, but patient outcomes are also affected by other disciplines, the severity and complexity of a patient's condition and the environment [17]. Previous systematic reviews, however, did not estimate the effect size of hospital factors. In this study, a pronounced effect of hospital characteristics and ward types on patient mortality was found. Taiwan hospitals are classified into three major types depending on accreditation: district, regional, and medical center. Generally, medical centers, with more critical care patients and a greater demand for complex technical skills, have a higher demand for advanced-skill nurses than either of the other two hospital types. Whether or not medical centers had significantly lower patient mortality rates than regional and district hospitals is influenced by the size effect and the effect of the hospital system complexity. The former effect means the quantity and quality of health care inputs in an academic medical center are much better than other types of hospitals, leading the medical center to association health outcome to be positive. The latter effect means that patients with the most complicated and most severe illness will be referred to a medical center for further medical treatment, leading the medical center to association of health outcome to be negative. Our finding indicates that there is no statistical difference in mortality between medical centers and district hospitals, a result revealing that the effect of the hospital system complexity should dominate the size effect in acute care hospitals in Taiwan.

A significant inverse relationship was found between nurse staffing levels and mortality. Evidence linking nurse staffing to patient mortality suggested that one way to improve quality is to increase nurse staffing [11,12,34]. Other studies, however, found that an all-RN staff was not strongly associated with patient outcomes [35,36]. One of the possible reasons is that nurse staffing variables include all nursing employees rather than just those who provide direct nursing care [37].

Nurse-staffing levels have been demonstrated in many studies, including this one, to be important in producing positive patient outcomes. In Taiwan, policymakers and hospital administrators have proposed many programs to lower turnover rate for nurses. These include retention programs and organizational restructuring focused on hospital managerial and operational efficiencies. Even with these changes, it is possible that hospitals faced with reduced payments tried to lower their operating costs-by reducing nursing staff, therefore, the basic problem behind the nursing shortage in Taiwan is most likely the inadequate nurse-to-patient ratios. Though the results of this study do not directly indicate an "ideal" nurse-to-patient ratio for Taiwan acute hospital wards and the economic value of adequate nurse-to-patient ratios, we believe the social benefit of providing better nurse staffing levels should be the driver in policy decisions. For Taiwanese policymakers, priority should be placed on mandating minimum nurse-to-patient ratios that will likely reduce patient mortality and lead to more manageable workloads, improve nurse job satisfaction and retention, and an amelioration of hospital nurse shortages. Though Taiwan already has such mandates, these current mandates are inadequate and lack enforcement. Furthermore, hospitals may not be in compliance with the ratio under current reimbursement schemes to hospitals made by the Taiwan NHI. The actual ratio is around one nurse to 10-11 patients in general medical-surgical wards for the day shift. In some Taiwanese hospitals, nurses on night shifts can be assigned up to 20-30 patients, five times the number of patients assigned to their European or U.S. counterparts [5]. As a patient safety intervention, this ratio seems to be unreasonable. We suggest that the DoH establish minimum, specific, and numerical licensed nurse-to-patient ratios by hospital ward on a shift-by-shift basis.

There are several further contributions of this study compared to previous studies. First, we conducted a longitudinal panel survey, so the reliability of this study should be higher than cross-sectional studies [38]. Second, our data are considered to be nationally representative of all hospitals in Taiwan. It follows that the threat to the external validity of this study is minor [38]. The results of this study could be generalized to the entire hospital population in Taiwan. Nevertheless, this study also has some limitations. First, assessment of severity of illness was limited, and may not be a valid reflection of acuity. Second, other factors influencing the nurse workload, such as number of admissions and discharges, may affect the relationship between staffing and patient outcomes and were not included in this study. Third, we used ward level data for our statistical analyses due to unavailability of patient level data. It should be addressed that ward level data were aggregated data from patients who hospitalized within the same ward. The findings of this study cannot be compared directly with a previous and most frequently cited study such as Aiken's study [10] because unit of analysis is different. The risk of death generated from Aiken's study was based on representative patient-level data, but our study estimates the risk of death based on a representative hospital ward-level data. It will result in the so-called ecological fallacy if one intends to derive some conclusion from a direct comparison of risk of death obtained from Aiken's study and the current study.

## Conclusions

Faced with the ever increasing pressure for hospitals to control costs while improving patient safety and quality, a shift-based minimum staffing ratio is imperative. Our analysis examined the relationship between nurse staffing and patient mortality. The results of this study can inform policymakers and hospital administrators about nurses' contribution to improving the quality of care. From a hospital's perspective, increased nurse staffing can be costly. Therefore, providing incentives such as funding and tax relief, and setting criteria for operation, licensure, and enforcement become two major options for dealing with health care issues. The government can consider providing payment supplements to hospitals to increase nurse staffing and bridge the gap between public and private valuation of this increased staffing. The central question needs to be dealt with cautiously as assurances are needed that funds provided to hospitals are actually used to increase nurse staffing. When taken together, by mandating minimum nurse-to-patient ratios, patients can have better nursing care and hospitals may avert preventable mortality and nurse shortages in hospital practice.

## Competing interests

The authors declare that they have no competing interests.

## Authors' contributions

YWL and WYC contributed to the conception of this paper, participated in its design and drafted the manuscript. WYC carried out the statistical procedures, interpretation of the data and wrote the manuscript. JLL carried out the data management and quality assurance of the data. LCH planned and supervised the project and gave advice concerning structuring the manuscript. All authors have seen and approved the final version. YWL and WYC had full access to all of the data in the study and take responsibility for the integrity of the data and the accuracy of the data analysis. All authors read and approved the final manuscript.

## Pre-publication history

The pre-publication history for this paper can be accessed here:

http://www.biomedcentral.com/1472-6963/12/44/prepub
